# Combining information from linkage and association mapping for next-generation sequencing longitudinal family data

**DOI:** 10.1186/1753-6561-8-S1-S34

**Published:** 2014-06-17

**Authors:** Brunilda Balliu, Hae-Won Uh, Roula Tsonaka, Stefan Boehringer, Quinta Helmer, Jeanine J Houwing-Duistermaat

**Affiliations:** 1 Department of Medical Statistics and Bioinformatics, Leiden University Medical Center, Einthovenweg 20, 2333 ZC Leiden, The Netherlands; 2 Netherlands Consortium for Healthy Ageing, Leiden University Medical Center, P.O. Box 9600, 2300 RC Leiden, The Netherlands

## Abstract

In this analysis, we investigate the contributions that linkage-based methods, such as identical-by-descent mapping, can make to association mapping to identify rare variants in next-generation sequencing data. First, we identify regions in which cases share more segments identical-by-descent around a putative causal variant than do controls. Second, we use a two-stage mixed-effect model approach to summarize the single-nucleotide polymorphism data within each region and include them as covariates in the model for the phenotype. We assess the impact of linkage disequilibrium in determining identical-by-descent states between individuals by using markers with and without linkage disequilibrium for the first part and the impact of imputation in testing for association by using imputed genome-wide association studies or raw sequence markers for the second part. We apply the method to next-generation sequencing longitudinal family data from Genetic Association Workshop 18 and identify a significant region at chromosome 3: 40249244-41025167 (p-value = 2.3 × 10^−3^).

## Background

In genetic association studies, joint analysis of multiple single-nucleotide polymorphisms (SNPs) can be more powerful than separate SNP analysis because single markers typically either have small effect sizes (common variants) or minor allele frequencies that are too small to reliably fit models (rare variants) [1]. If the rare variant effects were large, they would have been found through previous family-based linkage studies if the disease was not heterogeneous. There may be a middle ground in which multiple rare variants of moderate effect size play a key role in the etiology of some diseases. Such situations might be ideal for identity-by-descent (IBD) mapping [2]. Moreover, with the availability of genome-wide SNP data, the density of SNP markers has increased dramatically, making it possible to detect segments of IBD as small as 2 centimorgans (cM) [3].

In this article, we investigate the contribution that linkage-based methods, such as IBD mapping, can make to association mapping to identify rare variants in next-generation sequencing data. In the first part of our analysis, we use the methods of Browning and Thompson [2] to identify regions in which cases share more segments of IBD around a putative causal variant than do controls. After selecting these regions, we use a two-stage mixed-effects model approach, which was recently proposed by Tsonaka et al [4], to summarize the SNP data within each region and include them as covariates in the model for the phenotype. To increase our power to identify rare variants, we also include the number of rare variants per region as a covariate in the model.

To assess the impact of linkage disequilibrium (LD) on our analysis, we present results from estimating IBD probabilities using markers with and without LD. We assess the impact of imputation by analyzing both imputed dosage genome-wide association studies (DOS) and whole genome sequence (WGS) data. Table 1 provides a description of the data sets used for IBD and association mapping.

**Table 1:** Description of genotypic data sets used in each part of the analysis

Analysis	IBD mapping: regions with excess of IBD sharing		Association mapping: Two-stage approach	
**Name**	**AllMark**	**NoLD**	**DOS**	**WGS**

Type of data	GWAS (65,000, Illumina chips)		Imputed (dosage) WGS based on existing GWAS framework	Whole genome sequence
No. markers	~ 50,000	784	~1.2 million	~1.7 million
No. individuals	939		939	464

## Methods

### Study sample

We consider data from 939 individuals from 20 families; 464 are directly sequenced individuals and imputed WGS data, based on existing genome-wide association studies (GWAS) data, are available for their family members. We restrict our work to real genotypic data from chromosome 3. For each individual, we have information on age at examination and current tobacco smoking for up to 4 time points. We use the binary trait hypertension diagnosis at the first time point for selection of regions with excess IBD sharing and the quantitative trait diastolic blood pressure (DBP) for the phenotype model.

### Selection of regions with excess IBD sharing

We construct all possible case-case (CaCa) and case-control (CaCo) pairs, such that individuals within pairs are unrelated. This results in 9229 CaCa pairs and 10080 CaCo pairs. We estimate the IBD state using 2 data sets: one containing approximately 50,000 GWAS markers, which we refer to as the *AllMark *data set, and 1 containing only 784 LD-pruned GWAS markers, the *NoLD *data set. From both data sets we eliminate SNPs with minor allele frequencies (MAFs) <5% because shared alleles that are assumed to be rare represent strong evidence for IBD and can distort results if this assumption is violated [5]. In brief, the NoLD markers are selected using a sliding window 1 cM in size, removing markers based on linkage information content and excluding markers with the lowest MAF. At each marker we calculate the rate of IBD for each of the 2 groups and subtract their genomic average over all markers and pairs. If the ratio between CaCa pairs is larger than the maximum CaCo ratio, exceeding a certain threshold, we consider this region for association analysis.

To compute the IBD states between pairs of individuals, we use Thompson's method [6] with ibd_haplo (MORGAN) [6,7]. This method uses a continuous-time Markov rate matrix to model and estimate IBD states among pairs of individuals, using data at dense SNP loci, ignoring the LD structure. However, LD remains a major confounding factor because LD is itself a reflection of coancestry at the population level. To assess the impact of LD on IBD estimation, we present results for both AllMark and NoLD data sets.

In ibd_haplo, one needs to specify a value for parameters of the latent IBD process *β*, the pointwise pairwise probability of IBD, and α, the overall rate of change of IBD state along a chromosome. The choice of these parameters defines the time-depth of the IBD that is sought [5]. For the results shown in this paper, α = 0.05 and β = 0.01. We use a calling threshold of 0.9 as the probability that each of the IBD states must reach for the state to be called.

### Two-stage approach

After the regions have been selected, we use the two-stage approach of Tsonaka et al to test for their association with the longitudinal phenotype [4]. In the first stage, a random-effects model is used to summarize the regions via their empirical Bayes (EB) estimates. Next, the EB estimates of a specific region *r*, obtained from the first stage, are added as covariates into the model for the phenotype to test for region effects. Below, we describe in brief the phenotypic model used in the second stage.

Let *DBP*_ijt _be the diastolic blood pressure for individual *j *from family *i *at time point *t*, where *i *= 1, . . . , *N , j *= 1, . . . , n_i_, t = 1, . . . , 4, and n_i _is the number of individuals in family *i*. We use the following linear mixed model for each region *r*:

(1) $$DBP_{ijt}=\beta_{0}+\beta_{1}x_{ijt}+\beta_{2}eb_{ijr}+\beta_{3}s_{ijr}+u_{ij}+e_{ijt}$$

where ***x***_ijt _is the vector with covariate values for age and smoking status, *eb_ijr _*is the EB estimates of the region *r*, obtained from the first stage, and *s_ijr _*is the number of rare variants within region *r*; *u*_*ij*_ is the random family effect and ${\text{u}}_{i}=\left(u_{i1},\ldots ,u_{in_{i}}\right)$ follows a multivariate normal distribution with mean 0 and variance-covariance matrix $\sigma_{u_{i}}^{2}\times \text{R}$, where R is the coefficient of relationships matrix; *eijt *is a normally distributed residual with a 4 × 4 covariance matrix to model the correlation among 6 repeated measurements. We use a multivariate Wald statistic with 2 degrees of freedom to test the null hypothesis of no region effect; that is, *H0: β2 = β3 = 0*.

## Results

Table 2 presents the mean proportions and lengths of IBD segments shared for both groups. Averages were taken over all markers and all pairs. For both AllMark and NoLD, we observed a small difference in both mean proportion and length. However, in AllMark, where LD is ignored, the mean proportion of IBD is increased, as compared to NoLD. We compared the rates between the 2 groups and found 8 and 7 regions with an excess of IBD between CaCa pairs for AllMark and NoLD, respectively. Table 3 lists the starting and ending physical positions of these regions, as well as the number of SNPs and rare variants they contain. Interestingly, we observed no overlap between regions when using markers with and without LD.

**Table 2:** Description of IBD between case-case and case-control pairs

		**Mean proportions**			**Mean length of segments**		
			
**Data**	**Pairs**	**Any IBD**	**Not IBD**	**No call**	**Any IBD**	**Not IBD**	**No call**
			
AllMark	CaCa	0.295	0.499	0.206	58.27	144.48	25.98
	CaCo	0.292	0.503	0.205	58.01	145.58	25.91
NoLD	CaCa	0.006	0.950	0.044	44.81	316.00	21.27
	CaCo	0.004	0.951	0.045	39.52	315.09	21.59

**Table 3:** Descriptions of regions

**AllMark**					**NoLD**				
	
**Physical position**	**DOS**		**WGS**		**Physical position**	**DOS**		**WGS**	
	
**Start-end**	**N**	**n**	**N**	**n**	**Start-end**	**N**	**n**	**N**	**n**
	
27279401-27292557	77	38	100	61	29239664-29531222	2153	919	2984	1659
52618319-52637439	105	46	168	111	34834899-35282759	2730	1284	4267	2715
52759860-52771468	77	44	117	82	35718847-36018767	1618	927	2446	1755
52830547-52866115	291	156	379	244	36815704-37526013	3738	2151	5669	4038
86269515-86282586	60	24	96	58	40249244-41025167	4247	2530	6168	4214
99537305-99580268	211	120	322	260	167635899-168125439	2665	1349	3926	2552
99621002-99676384	270	144	386	299	168621773-168859006	1508	708	2018	1207
99927237-100004117	396	185	575	427					

N, number of SNPs per region; n, number of rare variants (MAF <5%) per region

After selecting the regions, we tested their association with the longitudinal phenotype by fitting a linear mixed model to DBP with the EB estimates per region, smoking status, and age as covariates. To further increase our power, we considered a second model, where we adjusted also for the sum of rare variants. We used 2 different genotype data, DOS with imputed genotypes on 939 individuals and WGS with complete genomics on 464 individuals. To account for multiple testing, we used a Bonferroni correction, using a significance level of alpha divided by the maximum number of independent regions tested for each data set; that is, 7 for the NoLD and 8 for the AllMark. We used 6 × 10^−3 ^as the significance level for AllMark and 7 × 10^−3 ^for NoLD.

No significant results were found when the candidate regions were selected using the AllMark data (results not shown). Table 4 gives the results of the analysis based on NoLD. When NoLD and DOS were used, there was a significant result for the region 3:40249244-41025167 (p-value of the 2df Wald 2.3 × 10^−3^). When WGS was used instead of DOS, the variance of the estimates increased and the signal was no longer significant. When the number of rare variants was removed from the model, the region again reached significance (p-value = 2.1 × 10^−3^).

**Table 4:** P-value for testing region effects using the NoLD data set

**DOS**				**WGS**			
	
**β2*^a^***	**β3*^a^***	**β2, β3*^a^***	**β2*^b^***	**β2*^a^***	**β3*^a^***	**β2, β3*^a^***	**β2*^b^***
	
0.03	0.25	0.04	0.02	0.04	0.76	0.12	0.04
0.93	0.91	0.99	0.92	0.81	0.27	0.54	0.93
0.99	0.11	0.27	0.77	0.35	0.41	0.50	0.51
0.18	0.24	0.25	0.20	0.32	0.13	0.15	0.23
1.3 × 10^−3^	0.05	2.3 × 10^−3^	3.6 × 10^−3^	9.3 × 10^−3^	0.33	0.01	2.1 × 10^−3^
0.29	1.00	0.55	0.22	0.27	0.75	0.54	0.28
0.09	0.66	0.22	0.09	0.25	0.26	0.31	0.33

Two different models are fitted: one with and one without including the number of rare variants as covariates. The regions are in the same order as in Table 3.

^a^Based on fitting ${\text{DBP}}_{ijt}=\beta_{0}+\beta_{1}x_{ijt}+\beta_{2}eb_{ijr}+\beta_{3}s_{ijr}+u_{i}+e_{ijt}$.

^b ^Based on fitting ${\text{DBP}}_{ijt}=\beta_{0}+\beta_{1}x_{ijt}+\beta_{2}eb_{ijr}+u_{i}+e_{ijt}$.

## Discussion

We have presented a method that combines linkage and association-based mapping to identify rare variants in next-generation sequencing data. Initially, we identify regions with an excess of IBD between case-case as compared to case-control pairs. Subsequently, we use a two-stage approach to summarize the regions via an EB estimate of the genetic variation and test for region effects. The two-stage approach captures the correlation between SNPs within regions by using random effects. These types of approaches can be more powerful than methods that ignore the dependency structure between the SNPs [8]. The approach can be directly applied to family and longitudinal data and can deal with missing genotypes.

One main advantage of this method, as compared to an association-only approach [9], is that by using the IBD mapping in the first step, we reduce the number of candidate regions to areas more enriched for putative causal loci. This considerably reduces the number of tests that need to be performed, and testing for interactions becomes feasible. This method can also be used for non-gene regions, although cautiously, because possibly important regions might already have been excluded in the first part, if the parameters for the IBD are misspecified. Moreover, if the resulting regions contain too many markers, the effect of rare variants might be diluted. The regions are selected using the binary hypertension diagnosis phenotype at the first measurement and not the quantitative DBP phenotype analyzed in the association study. This may be a problem if the 2 phenotypes are different. In our case, the binary phenotype was created using a threshold for the quantitative phenotype or information on medications. If the effect of a variant changes over time, we might lose power by determining the IBD states only on the first measurement. For individuals receiving treatment, the recorded DBP could be considered as a right-censored value, because we know that it is less than what the untreated value would be. Our approach ignores this information, which again may result in power loss. One way to address this issue could be to use a nonparametric algorithm to adjust blood pressure for treatment effect [10].

In this article, we do not present results for type I error or power. However, Tsonaka et al. [4] and Houwing-Duistermaat et al. [9] report results for both regarding the two-stage approach. Using extensive simulations, Tsonaka et al. showed that the test statistics preserve the type I error at nominal level for scenarios comparable to ours. Houwing- Duistermaat et al. analyzed the simulated phenotypes from this Genetic Analysis Workshop (GAW) and found that the power was as high as 96.5% and 72.5% using the imputed GWAS and WGS data, respectively.

We found significant results only when the candidate regions were selected using the NoLD and DOS data. One reason for the better performance of the NoLD data, as compared to the AllMark data, could be the presence of LD in the latter. LD leads to increased rates of false positive IBD results [5], which could erroneously indicate these regions as interesting. The absence of overlap between regions when using these 2 data sets also indicates the sensitivity of the method to the amount of LD in the data. Another reason for the better performance of the NoLD data set could be the region selection process. In the NoLD data, the markers are further apart from each other, as compared to the AllMark data set. Hence, when selecting a region (at least 2 markers), we automatically include more SNPs and rare variants.

When the NoLD and WGS data were used, the signal of the region found using DOS was no longer significant. This power loss could be a result of the smaller sample size in the WGS data, which leads to increased variances of the parameter estimates (results not shown). The same happens for the estimates of the genetic variance. On one hand, using the DOS data we estimate $\sigma_{u}^{2}=10.622$ with a variance of 1.4366 (p-value 6.9 × 10^−11^). On the other hand, when using WGS, the estimate becomes much smaller, $\sigma_{u}^{2}=1.153$, and its variance increases to 27.23 (p-value = 0.99). Removing the number of rare variants from the model led to a significant p-value for this region.

Using the NCBI database [11], we found that the gene *CADM2*, which is 146 kilobase (kb) on the right of the region we identified, is associated, among other phenotypes, with blood pressure and body mass index [12]. More specifically, 3 SNPs in this gene are associated with blood pressure: rs1370032 (p-value = 7.22 × 10^−5^), rs13074417 (p-value = 7.625 × 10^−5^), and rs4859048 (*p*- value = 7.872 × 10^−5^).

## Conclusions

We identified a significant region when IBD states were determined using LD-pruned markers and association with phenotype was tested using the imputed genotypes of 939 individuals. When the raw sequence data of 464 individuals was used, the signal was significant only after the number of rare variants from the phenotype model were removed.

## Competing interests

The authors declare that they have no competing interests.

## Authors' contributions

BB participated in the design of the study, performed the statistical analysis, interpreted the results, and wrote the manuscript. HWU participated in the design of the study and in the interpretation of the results. RT developed the statistical method. SB participated in the design of the study and helped to draft the manuscript. QH participated in the analysis of the data and the interpretation of the results. JJHD conceived of the study, participated in its design and coordination, and helped draft the manuscript. All authors read and approved the final manuscript.
